# The role of teachers in the prevention and management of childhood obesity among school-aged children in Ghana: a cross-sectional study

**DOI:** 10.3389/fpubh.2025.1724617

**Published:** 2025-12-03

**Authors:** Chantal Kayitesi, Nadia Tagoe, Princess Ruhama Acheampong

**Affiliations:** 1 Department of Global and International Health, School of Public Health, Kwame Nkrumah University of Science and Technology, Kumasi, Ghana; 2 Department of Health Promotion and Disability Studies, School of Public Health, Kwame Nkrumah University of Science and Technology, Kumasi, Ghana

**Keywords:** childhood obesity, school-based interventions, teachers, public health, Ghana

## Abstract

**Background:**

School-based interventions have been widely used in the prevention and control of childhood obesity. Teachers, as pivotal figures in child development, are strategically situated to aid in this process. Nonetheless, there is limited evidence regarding their role, awareness, and contribution to childhood obesity in Ghana. This study evaluated the role of teachers in the prevention and management of childhood obesity in Ghana.

**Materials and methods:**

This quantitative, cross-sectional study included 74 teachers selected through convenience sampling from public schools in the Oforikrom Municipality of Kumasi, the capital of Ghana’s Ashanti Region. Data were gathered using a structured pre-tested questionnaire and analyzed using the R programming software. Binary logistic regression was performed to identify the factors associated with teacher’s participation in childhood obesity prevention programs.

**Results:**

The study revealed that 46% of teachers were familiar with childhood obesity with only 33.8% of teachers familiar with its causes. Significant institutional hurdles included resource restrictions (46%), insufficient training (24.3%), and time constraints (12.2%). Chi-square analysis revealed that teachers’ participation in childhood obesity prevention was significantly associated with socioeconomic conditions (*p* = 0.016), challenges faced (*p* = 0.022), perception of physical activity (*p* = 0.012), physical education practices (*p* = 0.006), outdoor play promotion (*p* = 0.026), health screenings (*p* = 0.002), healthy eating education (*p* = 0.002), and counseling practices (*p* < 0.001). However, the multivariable logistic regression analysis revealed no statistically significant determinants of teachers’ participation.

**Conclusion:**

The results highlight the necessity for specific measures, including professional development initiatives, institutional policy modifications, and resource distribution, to improve teachers’ ability to combat childhood obesity. Policymakers ought to incorporate comprehensive school health initiatives into the curriculum, prioritizing physical exercise and nutritious food.

## Introduction

Obesity is a significant risk factor for non-communicable diseases (NCDs), including hypertension, cardiovascular illnesses, and certain malignancies (1). Over the last four decades, obesity has intensified into a global public health emergency, substantially exacerbating the prevalence of non-communicable diseases worldwide (2). The World Health Organization (WHO) categorizes overweight as a body mass index (BMI) of 25–29 kg/m^2^ and obesity as a BMI of 30 kg/m^2^ or more (3). Current figures indicate that 38% of the global population is classified as overweight or obese, with forecasts suggesting a significant rise in prevalence by 2030 (4, 5).

Childhood obesity is increasing, with more than 340 million children and adolescents aged 5–19 classified as overweight or obese in 2020, alongside 39 million children under 5 years old (6). Low- and middle-income countries (LMICs) have experienced a swift rise in obesity prevalence. In Tanzania, childhood obesity among school-aged children aged 6–9 ranges from 5.6% in Dodoma to 6.3% in Kinondoni (7). In Ghana, childhood obesity is an emerging public health issue, particularly in the urban areas (8) with 5% of school children in Cape Coast considered as obese, an additional 9% at high risk (9), and 2.3% in Kumasi, considered overweight and obese (10). Contributing factors include urbanization, industrialization, dietary shifts, and sedentary lifestyles (11, 12).

School-based interventions such as nutrition programs, physical activity promotion, and behavioral change initiatives have demonstrated potential in LMICs (13). These strategies aim to enhance nutritional status and promote healthier behaviors in children by utilizing schools as delivery platforms (14). Successful implementation requires collaboration with teachers, who are essential partners in executing effective obesity prevention measures. Teachers recognize the importance of health promotion in schools, as healthy students often achieve superior academic outcomes (15). Despite this awareness, societal prejudices and classroom-based constraints limit their effectiveness (16). Engaging teachers in program development improves both ownership and impact (17).

The Ghana Education Service (GES) introduced the School Health Education Programme (SHEP) in 1992 to promote health education in schools, aiming to equip students with life skills for healthy behaviors (18). However, evidence suggests that teachers often lack the training, knowledge, and resources necessary to address childhood obesity (9, 19). Although studies have been conducted to assess the role of teachers internationally (15, 17), limited research has been carried out in Ghana. Given the increasing rates of childhood obesity in Ghana and the essential role of teachers, this study seeks to assess their understanding of childhood obesity, available resources, policy frameworks, and the challenges they face. The findings will help develop targeted interventions and capacity-building programs for teachers in the country, with the broader aim of reducing childhood obesity rates, improving health outcomes for school-aged children, and supporting Ghana’s efforts to curb NCDs in its young population. This study therefore assessed the role of teachers in preventing and managing childhood obesity and identified the barriers and facilitators affecting their participation in obesity prevention among school-aged children in Kumasi, Ghana.

## Materials and methods

### Study design

Using a quantitative approach, we employed a cross-sectional study design to assess teachers’ awareness, challenges, and strategies in childhood obesity prevention in Ghana.

### Study setting

The study was conducted in selected schools within the Oforikrom Municipality, Kumasi in the Ashanti Region of Ghana (between longitude 6.35°–6.40° and longitude 1.30°–1.35°). The municipality covers a land area of 54.1 km^2^ with a population of 303,016 people with a population density of 5,601 persons per km^2^. This urban municipality encompasses diverse educational institutions, providing a representative environment to study the role of teachers in managing childhood obesity. There are 24 public schools with 410 teachers in basic or primary and junior high schools under the Ghana Education Service in Oforikrom Municipal.

### Study population and sampling

The target population is comprised of teachers in basic (lower primary, upper primary), and junior high schools within the municipality. The convenience sampling method was used to recruit participants due to time and logistical constraints, allowing the inclusion of teachers that were readily accessible. Teachers at these levels were deemed appropriate respondents due to their daily interactions with school-aged children and their potential role in childhood obesity prevention.

### Inclusion and exclusion criteria

The study included teachers actively engaged in teaching at the time of data collection, as well as those who were willing to participate and thus provided informed consent. Teachers on leave or who were absent during the data collection period were excluded from the study.

### Sample size determination

A sample size of 74 teachers was used in this study. This size was obtained using a power-based approach to detect a 20% difference in the proportion of teachers participating in childhood obesity prevention initiatives based on awareness (40% for aware teachers vs. 20% for non-aware teachers). The calculation assumed a 95% confidence level (*α* = 0.05, two-sided test), 70% power (*β* = 0.30), and a standard normal approximation for proportions (20). The relatively lower power was chosen due to the limited population of available teachers and the exploratory nature of the study. Standard normal approximations for proportions were used and no design effect or continuity correction was applied (20).

### Data collection

A structured questionnaire, which included both closed-ended and open-ended questions, was developed to obtain demographic details, awareness, and perceptions of childhood obesity. Data on institutional strategies toward prevention and challenges and facilitators to teachers’ participation in childhood obesity prevention were also collected (Supplementary File 1).

The demographic details captured included age, gender, educational level, teaching experience, and grade level taught. The second section assessed teachers’ awareness and perceptions of childhood obesity through items on familiarity with obesity and its causes, recognition of associated health risks (e.g., type 2 diabetes, hypertension, and psychological effects), and sources of information. The third section examined school-level systems and prevention strategies, with items assessing whether schools had policies on healthy eating, physical activity programs, healthy snack initiatives, or nutritional education activities. The final section explored barriers and facilitators to teachers’ participation in childhood obesity prevention and management, including items on challenges such as limited resources, lack of training, time constraints, and parental support. Enabling factors such as allocated time for health education, professional training, curriculum support, and collaboration with healthcare staff were also captured.

The questionnaires were self-administered by study participants in selected schools, with teachers spending about 30 min to complete them. Data were collected over a four-week period. Respondents were given sufficient time to complete the questionnaire, and all forms were completed on-site to ensure data integrity.

### Ethics approval

Ethical approval (CHRPE/AP/983/24) for the study was obtained from the Committee for Human Research and Publication Ethics (CHRPE), at the Kwame Nkrumah University of Science and Technology (KNUST).

### Questionnaire validity and reliability

Reliability analysis was conducted to assess the internal consistency of the items in the questionnaire using Cronbach’s alpha. The calculated Cronbach’s alpha was 0.83, suggesting questionnaire reliability in consistently assessing the study objectives.

### Data management and analysis

Data were cleaned and managed using Microsoft Excel 2019 and analyzed using R programming statistical software (R 4.4.1), at a significance level set at *p* < 0.05 and a confidence level set at 95%. Descriptive statistics such as frequencies and percentages were used to summarize the distribution of study variables. Inferential statistics, such as the chi-square test or Fischer’s exact test, were employed to examine associations between demographic and other teacher characteristics and their participation toward the prevention and management of childhood obesity in schools. Univariable and multivariate binary logistic regression analyses were also utilized to identify the independent predictors that influence teacher’s participation to prevent and manage childhood obesity.

## Results

### Sociodemographic characteristics of study participants

The socio-demographic characteristics of the 74 teachers involved in the study are summarized in Table 1. Many participants were aged between 31 and 40 years (43.2%), followed by those aged 20–30 years (35.1%). Females made up most of participants (64.9%), while males accounted for 35.1%. The vast majority of participants (82.4%) held a bachelor’s degree, while 9.5% had a Diploma, and 8.1% had attained a master’s degree. A substantial proportion (40.5%) had 0–5 years of teaching experience. Participants with 6–10 years of experience accounted for 17.6%, 11–15 years for 20.3%, and those with more than 15 years made up 21.6%. Most participants (58.1%) taught at the Junior High School level, followed by Upper Primary (27.0%) and Lower Primary (14.9%) (Table 1).

**Table 1:** Socio-demographic characteristics of study participants.

Variable	Frequency (*n* = 74)	Percentage (%)
Age group (years)		
20–30	26	35.1
31–40	32	43.2
41–50	10	13.5
Above 50	6	8.1
Gender		
Female	48	64.9
Male	26	35.1
Educational level		
Diploma	7	9.5
Bachelor’s Degree	61	82.4
Master’s Degree	6	8.1
Teaching experience		
0–5 years	30	40.5
6–10 years	13	17.6
11–15 years	15	20.3
More than 15 years	16	21.6
Grade level taught		
Lower primary	11	14.9
Upper primary	20	27
Junior high school	43	58.1

### Level of awareness of childhood obesity among teachers

Findings on teachers’ awareness of childhood obesity, associated health risks, and sources of information are presented in Table 2. Most teachers reported being familiar with being overweight and obesity, with 44.6% indicating they were “familiar” and 40.5% “familiar to some extent.” Similarly, 46% were “familiar” with childhood obesity, although 14.9% reported no familiarity. Awareness of the causes of obesity was generally moderate, with 39.2% “familiar to some extent” and 33.8% “familiar,” while 16.2% were not familiar and 1.4% were unsure. Regarding associated health risks, 63.5% recognized type 2 diabetes as a potential consequence of childhood obesity, while 62.2% identified hypertension or heart disease. Awareness of psychological risks was relatively lower, with 48.7% acknowledging this association. The most common source of information on childhood obesity was the school setting (52.7%), followed by social media (24.3%), while few cited hospitals, community gatherings, or print media (Table 2).

**Table 2:** Awareness of childhood obesity, associated risks, and information sources among teachers.

Awareness characteristic	Frequency (*n* = 74)	Percentage (%)
Familiarity with overweight/obesity		
Extremely familiar	6	8.1
Familiar	33	44.6
Familiar to some extent	30	40.5
Not familiar	5	6.8
Familiarity with childhood obesity		
Extremely familiar	3	4.1
Familiar	34	46
Familiar to some extent	26	35.1
Not familiar	11	14.9
Familiarity with causes of obesity		
Extremely familiar	7	9.5
Familiar	25	33.8
Familiar to some extent	29	39.2
Not familiar	12	16.2
Not sure	1	1.4
Awareness of risk: type 2 diabetes		
No	4	5.4
Not sure	16	21.6
Yes	47	63.5
Unknown	7	9.5
Awareness of risk: hypertension/heart disease		
No	8	10.8
Not sure	15	20.3
Yes	46	62.2
Unknown	5	6.8
Awareness of psychological risks		
No	16	21.6
Not sure	17	23
Yes	36	48.7
Unknown	5	6.8
Sources of information		
Never heard about it	1	1.4
Church	1	1.4
Durbar	1	1.4
Hospital	7	9.4
News paper	1	1.4
School	39	52.7
Social media	18	24.3
Social media & News paper	1	1.4
Unknown	5	6.7

### Perceptions of childhood obesity as a public health issue

**Figure 1:**
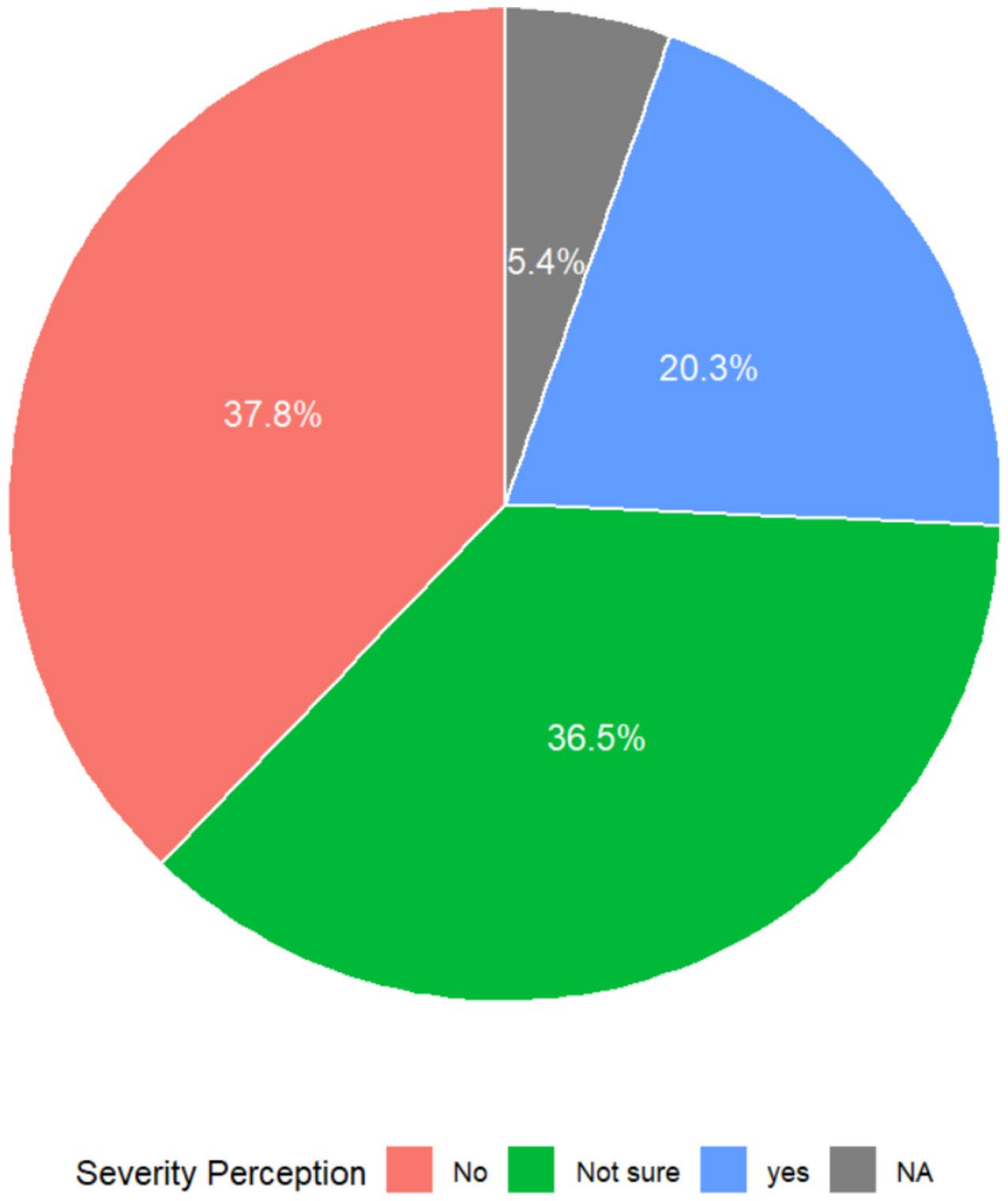
Perceptions of childhood obesity as a public health issue.

Respondents’ perceptions regarding childhood obesity as a public health issue are illustrated in Figure 1. About 37.8% of the respondents did recognize childhood obesity as a public health issue while 36.5% were unsure. Only 20.3% of respondents recognized childhood obesity as a public health concern, highlighting a minority that indicates potential health risks and societal impact (Figure 1).

### Institutional systems and strategies

The institutional systems and strategies implemented in schools to address childhood obesity among school-aged children are summarized in Table 3. The presence of school policies promoting healthy eating and physical activity was limited, with 67.6% of participants indicating the absence of such policies. Few teachers reported having these policies (14.9%), while 17.6% were unsure about their existence. Over half (54.1%) of the participants indicated that their schools lacked initiatives that were targeted at addressing childhood obesity, and 20.3% were uncertain about their implementation. Only a quarter (25.6%) of participants acknowledged having these initiatives in place. Physical activity programs were available in all participating schools, with 73.0% reporting the presence of such programs. However, 16.2% reported that such programs were not implemented in their schools, and 10.8% were unsure about their availability. Healthy diets programs were reported to be present by 60.8% of teachers. However, 18.9% of participants reported the absence of healthy diet programs in their schools (Table 3).

**Table 3:** Institutional systems and strategies for preventing and managing childhood obesity in schools.

Characteristic	Frequency (*n* = 74)	Percentage (%)
School policies on healthy eating and physical activity		
Yes	11	14.9
No	50	67.6
Not sure	13	17.6
School initiatives on health snacks and beverages		
Yes	19	25.6
No	40	54.1
Not sure	15	20.3
Availability of physical activity program		
Yes	54	73.0
No	12	16.2
Not sure	8	10.8
Availability of healthy diet program		
Yes	45	60.8
No	14	18.9
Not sure	15	20.3

### Types of preventive measures available in schools

**Figure 2:**
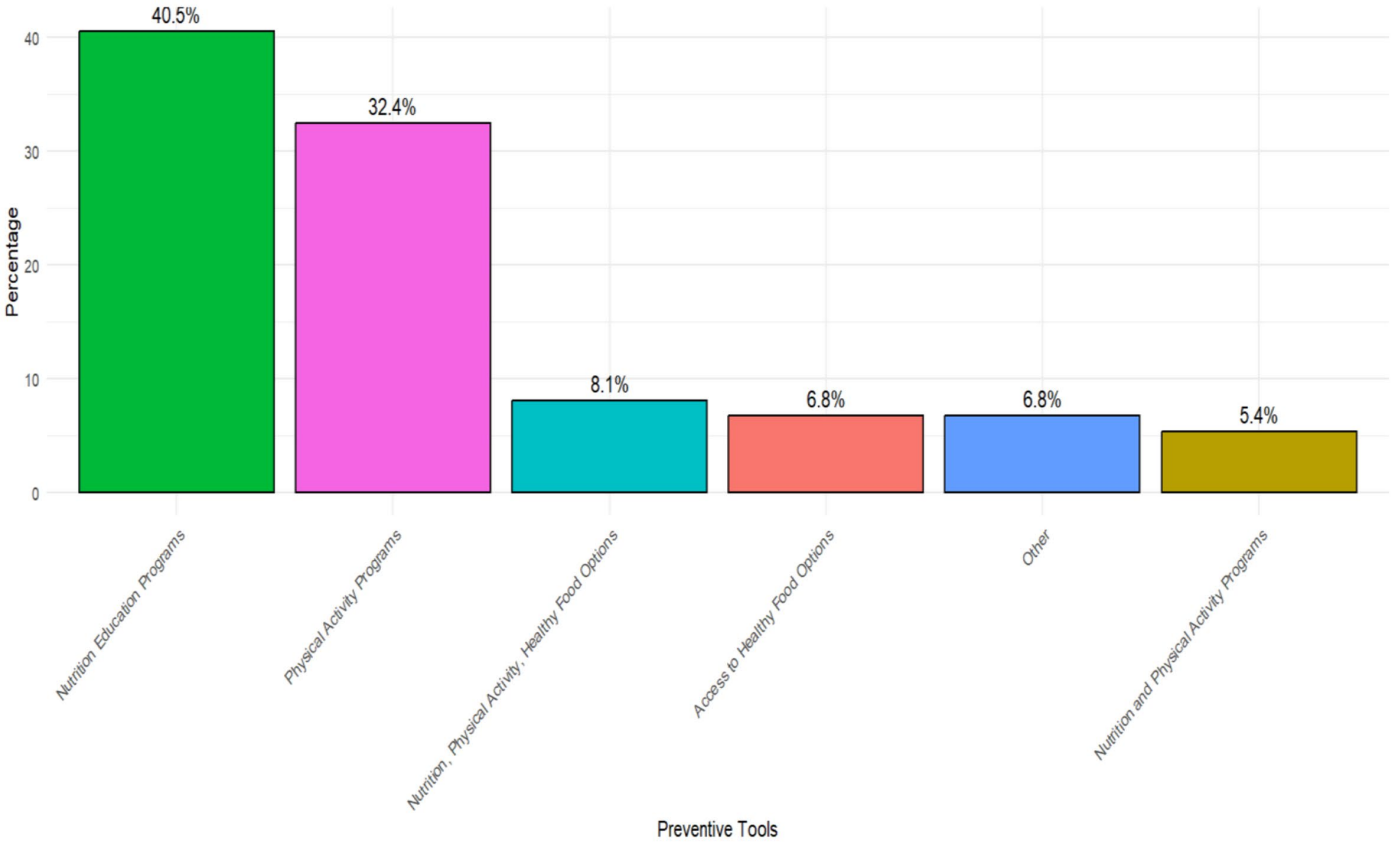
Types of preventive strategies available in schools to address childhood obesity.

The study identified various preventive measures implemented in schools to address childhood obesity (Figure 2). Nutritional education programs were the most frequently reported measure, implemented by 40.5% of participants with physical activity programs accounting for 32.4%. Nutrition, physical activity, and healthy food options, as a comprehensive approach combining multiple strategies, were reported by 8.1% of participants. 6.8% of teachers reported that efforts were made to provide nutritious food alternatives within the school environment. An integrated approach combining nutrition and physical activity programs was implemented in 5.4% of schools. Miscellaneous measures, reported by 6.8% of teachers, included less conventional or unspecified interventions (Figure 2).

### Hindrances and facilitators to teachers’ participation in prevention and management of childhood obesity

The key factors that hindered teachers’ participation in the prevention and management of childhood obesity are presented in Table 4. Inadequate resources emerged as the most common barrier, reported by 46% of respondents. A lack of training was also notable, cited by 24.3% of teachers, while 12.2% indicated insufficient time as a constraint. Additionally, 10.8% reported a lack of parental support, and 2.7% pointed out limited support from school administration (Table 4).

**Table 4:** Summary of challenges faced by teachers.

Challenge	Frequency (*n* = 74)	Percentage (%)
Inadequate resources	34	46.0
No parental support	8	10.8
No school administration support	2	2.7
No training	18	24.3
Not enough time	9	12.2

### Factors facilitating teachers’ participation in prevention and management of childhood obesity

**Figure 3:**
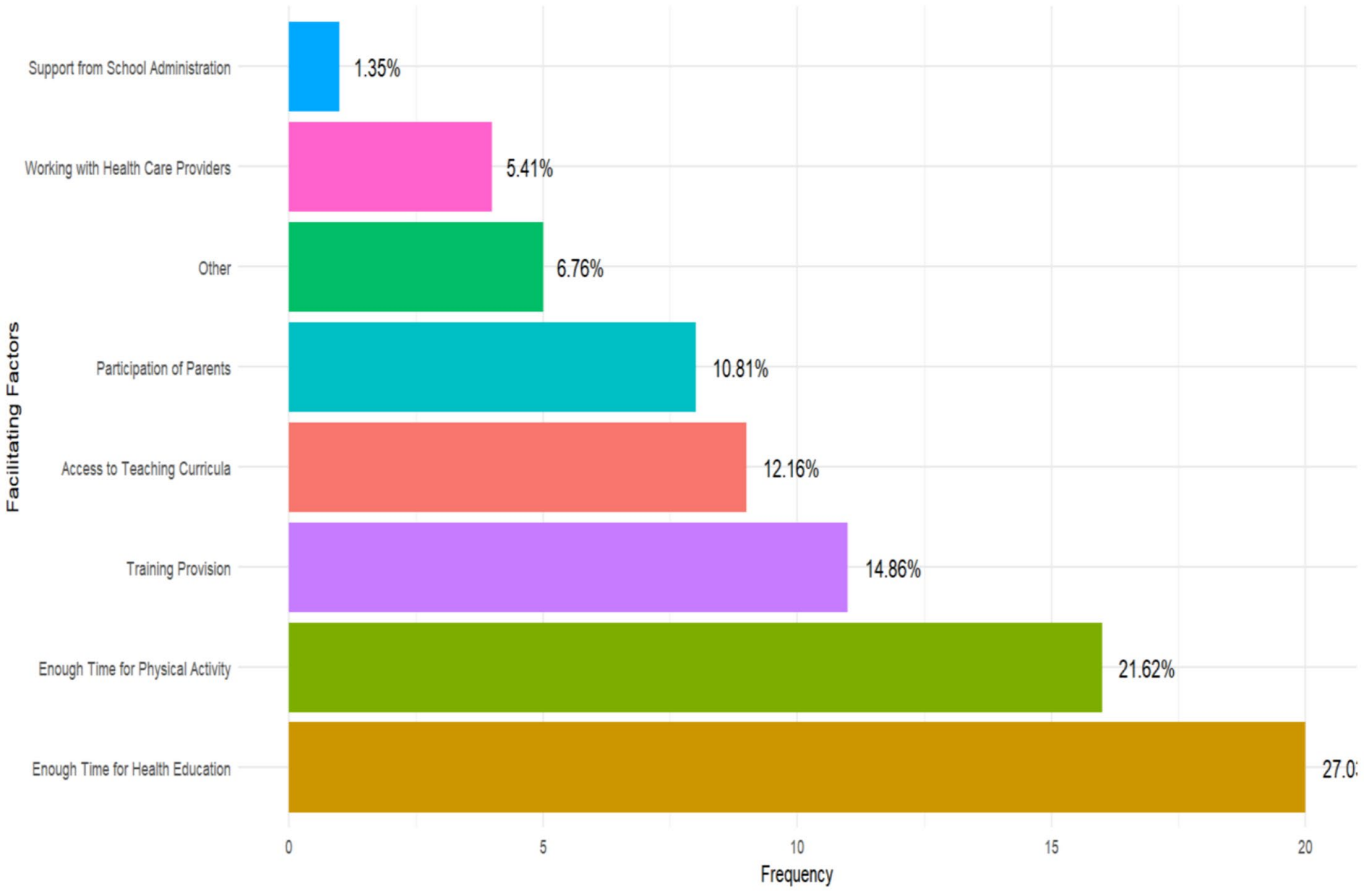
Factors facilitating teachers’ participation in prevention and management of childhood obesity.

The various factors that facilitated teachers’ involvement in addressing childhood obesity prevention and management are summarized in Figure 3. Overall, relatively fewer teachers reported the existence of facilitators. The most frequently reported facilitator was having adequate time allocated for health education and physical activity, with only 27.0 and 21.6% of teachers identifying these as key enabling factors, respectively. Professional training was identified by 14.9% of teachers as a critical facilitator, with a smaller proportion of teachers (12.2%) emphasizing the importance of having access to structured teaching curricula. Other facilitators included parental participation (10.8%), working with healthcare workers (5.41%), and support from the school administration (1.35%) (Figure 3).

### Association between teacher characteristics, challenges and type of prevention practice and participation in childhood obesity prevention

A chi-square analysis examining the factors associated with teachers’ participation in the prevention and management of childhood obesity is presented in Table 5. Awareness status, age group, gender, educational level, and perception of healthy eating were not significantly associated with participation (*p* > 0.050). However, teachers’ socioeconomic conditions were significantly associated (*p* = 0.016), with a higher proportion of participants reporting no socioeconomic influence (39%) compared to non-participants (5.6%).

**Table 5:** Association between teachers’ characteristics, challenges and prevention practices with participation in obesity prevention programs.

Variable	Does not participate (*n* = 21)	Participates (*n* = 53)	*p*-value
Awareness status			>0.999
Not aware	3 (14%)	8 (15%)	
Aware	18 (86%)	45 (85%)	
Age group			0.500
20–30 years	5 (24%)	21 (40%)	
31–40 years	12 (57%)	20 (38%)	
41–50 years	3 (14%)	7 (13%)	
Above 50 years	1 (4.8%)	5 (9.4%)	
Gender			0.068
Female	17 (81%)	31 (58%)	
Male	4 (19%)	22 (42%)	
Educational level			0.600
Diploma	2 (9.5%)	5 (9.4%)	
Bachelor’s Degree	16 (76%)	45 (84.9%)	
Master’s Degree	3 (14%)	3 (5.7%)	
Socioeconomic influence			**0.016**
No	1 (5.6%)	19 (39%)	
Not sure	9 (50%)	11 (22%)	
Yes	8 (44%)	19 (39%)	
Challenges faced			**0.022**
Lack of support	2 (9.5%)	8 (15%)	
Lack of training	10 (48%)	8 (15%)	
Other	1 (4.8%)	2 (3.8%)	
Resource-related issues	8 (38%)	26 (49%)	
Time constraints	0 (0%)	9 (17%)	
Prevention via physical activity			**0.012**
No	1 (5.3%)	6 (12%)	
Not sure	8 (42%)	5 (10%)	
Yes	10 (53%)	39 (78%)	
Prevention via healthy eating			0.500
No	0 (0%)	1 (2.0%)	
Not sure	3 (16%)	3 (6.0%)	
Yes	16 (84%)	46 (92%)	
Physical education practices			**0.006**
Always	2 (10%)	4 (7.7%)	
Frequently	2 (10%)	24 (46%)	
Never	3 (15%)	2 (3.8%)	
Rarely	4 (20%)	2 (3.8%)	
Sometimes	9 (45%)	20 (38%)	
Outdoor play practices			**0.026**
Always	4 (21%)	9 (17%)	
Frequently	3 (16%)	21 (40%)	
Never	3 (16%)	0 (0%)	
Rarely	0 (0%)	1 (1.9%)	
Sometimes	9 (47%)	22 (42%)	
Health screening practices			**0.002**
Always	0 (0%)	4 (8.2%)	
Frequently	0 (0%)	5 (10%)	
Never	8 (42%)	5 (10%)	
Rarely	8 (42%)	9 (18%)	
Sometimes	3 (16%)	26 (53%)	
Healthy eating education practices			**0.002**
Always	2 (10.6%)	6 (11.5%)	
Frequently	1 (5.3%)	11 (21%)	
Never	6 (32%)	2 (3.8%)	
Rarely	5 (26%)	4 (7.7%)	
Sometimes	5 (26%)	29 (56%)	
Counseling practices			**<0.001**
Always	0 (0%)	14 (27.9%)	
Frequently	2 (11%)	21 (41%)	
Never	4 (22%)	0 (0%)	
Rarely	6 (33%)	4 (7.8%)	
Sometimes	6 (33%)	12 (24%)	
Material availability for obesity prevention			0.500
No	18 (86%)	37 (71%)	
Not sure	3 (14%)	9 (17%)	
Yes	0 (0%)	6 (11.5%)	

Data presented as frequency and percentage. *p* values computed using the chi-square test analysis. Bolded *p* values indicate statistical significance.

Significant associations were also observed between participation and several challenges, including lack of training, time constraints, and resource-related issues (*p* = 0.022). Perceptions of physical activity as a preventive strategy were significantly associated with participation (*p* = 0.012), with 78% of participants endorsing it compared to 53% of non-participants. Similarly, physical education practices (*p* = 0.006), outdoor play (*p* = 0.026), health screening (*p* = 0.002), healthy eating education (*p* = 0.002), and counseling practices (*p* < 0.001) showed significant associations with participation. No significant association was found between the availability of obesity prevention materials and participation (*p* = 0.500) (Table 5).

### Predictors for teachers participation in childhood obesity prevention programs

The univariable and multivariable logistic regression analyses of predictors for participation in childhood obesity prevention programs is illustrated in Table 6. At the univariable level, teachers who were unsure about the influence of their socioeconomic conditions were significantly less likely to participate (cOR = 0.06, 95% CI: 0.01–0.58, *p* = 0.014), though this association was not significant after adjustment (aOR = 0.10, 95% CI: 0.00–1.28, *p* = 0.100). A similar trend was observed among those uncertain about the role of physical activity (cOR = 0.10, *p* = 0.064; aOR = 0.05, *p* = 0.150). Frequent engagement in physical education and healthy eating practices was associated with higher odds of participation, but none reached statistical significance in the adjusted models. Overall, no predictors remained statistically significant after adjusting for cofounding factors (Table 6).

**Table 6:** Univariable and multivariable logistic regression analysis of statistically significant predictors for participation in childhood obesity prevention programs.

Variable	cOR (95% CI)	*p*-value	aOR (95% CI)	*p*-value
Socioeconomic influence				
No	Ref		Ref	
Not Sure	0.06 (0.01–0.58)	**0.014**	0.10 (0.00–1.28)	0.100
Yes	0.13 (0.01–1.10)	0.061	0.47 (0.01–17.8)	0.700
Challenges faced				
Lack of Support	Ref			
Lack of Training	0.20 (0.03–1.22)	0.081	—	—
Resource-related Issues	0.81 (0.14–4.63)	0.815	—	—
Time Constraints	—	0.999	—	—
Other	0.50 (0.03–8.71)	0.634	—	—
Prevention via physical activity				
No	Ref		Ref	
Not Sure	0.10 (0.01–1.14)	0.064	0.05 (0.00–1.73)	0.150
Yes	0.65 (0.07–6.03)	0.705	0.26 (0.00–16.5)	0.500
Physical education practices				
Always	Ref		Ref	
Never	0.33 (0.03–3.93)	0.383	3.27 (0.06–220)	0.600
Rarely	0.25 (0.02–2.76)	0.258	0.35 (0.01–11.7)	0.600
Sometimes	1.11 (0.17–7.22)	0.912	1.87 (0.09–52.0)	0.700
Frequently	6.00 (0.65–55.66)	0.115	12.4 (0.26–1.42)	0.200
Outdoor play practices				
Always	Ref			
Never	—	0.994	—	—
Rarely	—	0.997	—	—
Sometimes	1.09 (0.27–4.45)	0.908	—	—
Frequently	3.11 (0.57–16.83)	0.188	—	—
Health screening practices				
Always	Ref			
Never	—	0.997	—	—
Rarely	—	0.997	—	—
Sometimes	—	0.997	—	—
Frequently	—	1.000	—	—
Healthy eating education practices				
Always	Ref		Ref	
Never	0.08 (0.01–1.26)	0.073	0.18 (0.00–14.0)	0.400
Rarely	0.20 (0.02–2.58)	0.217	0.33 (0.00–27.3)	0.600
Sometimes	1.45 (0.13–15.79)	0.760	6.40 (0.10–46.0)	0.400
Frequently	2.75 (0.14–55.17)	0.508	12.9 (0.11–26.96)	0.300
Counseling practices				
Always	Ref			
Never	—	0.995	—	—
Rarely	—	0.995	—	—
Sometimes	—	0.995	—	—
Frequently	—	0.996	—	—

cOR: crude odd ratio; aOR: adjusted odd ratio; CI: confidence interval. *p* < 0.05 bolded and considered statistically significant.

## Discussion

The increase in childhood obesity worldwide has emerged as a critical public health issue. School-based programs have long been central to childhood obesity prevention, with efforts focused on promoting physical activity, providing nutrient-dense food options, and offering nutrition education. Schools provide a unique setting for health promotion, as they have direct access to children during their formative years, where lifelong habits are established. Teachers are thus pivotal in integrating nutrition education into the curriculum, promoting physical activity, and fostering a supportive school environment. However, limited research exists in Ghana on teachers’ actual participation and the factors influencing their engagement in school-based obesity prevention efforts. This study assessed the role of teachers in the prevention and management of childhood obesity among school aged children in Kumasi, Ghana.

The findings indicate that teachers generally possessed a high level of familiarity with childhood obesity, its related health risks and causes. Additionally, there was significant awareness of the health risks related to childhood obesity, specifically type 2 diabetes, hypertension, and heart disease among the teachers. These findings align with prior research indicating that teachers recognize these risks, potentially influenced by media campaigns and public health communications. The lower awareness of the psychological risks associated with obesity (52%) indicated a knowledge gap that future training programs for teachers should address (21). The psychological effects, among which are low self-esteem, depression, and stigmatization, are commonly not emphasized in public discussions, but these contribute significantly to the overall development of a child. Addressing this gap might better enable teachers to provide students with holistic support and advocate for more comprehensive school-based health efforts (22).

A notable percentage of respondents (37.8%) did not perceive childhood obesity as a public health concern, while 36.5% expressed uncertainty. This ambiguity in perception underscores a possible deficiency in comprehending the gravity of childhood obesity as a public health concern. The minimal number (20.3%) of respondents identifying it as a significant concern may be associated with widespread underappreciation of the long-term ramifications of pediatric obesity, including an elevated risk for chronic diseases (23). The results are troubling, indicating that many teachers lack comprehensive awareness of the overall public health challenges of childhood obesity, potentially obstructing effective prevention initiatives in schools. There is therefore the need for more focused initiatives designed to alter teachers’ attitudes toward juvenile obesity as a critical health problem. Training workshops, curriculum enhancements, and collaboration with health professionals could play a crucial role in shaping more informed attitudes and encouraging proactive involvement. The failure to acknowledge pediatric obesity as a public health concern is not exclusive to Ghana; similar trends have been noted in other LMICs, where health education frequently lacks emphasis in the curriculum (24–26). This is indicative of a global institutional issue where rampant communicable diseases in LMICs are often prioritized over the growing non-communicable health challenges such as childhood obesity. Moreover, non-communicable diseases (NCDs) are often associated with adults and the people over 65, despite their increasing prevalence in children, which contributes to missed opportunities for prevention and control among younger populations (27). Addressing this gap will require national policy changes to make health promotion a central core element of educational systems.

The research revealed that institutional frameworks and strategies for the prevention and management of childhood obesity in schools were inadequate. Most participants indicated that their schools did not have policies to encourage healthy food and physical activity. These findings reflect widespread concerns over the absence of comprehensive, school-wide health measures to address childhood obesity (28, 29). The limited occurrence of efforts advocating healthy snacks and physical activity programs emphasize the necessity for more institutional support for obesity prevention. These findings are similar to reports from other studies conducted in other African schools, where resources and institutional support for obesity prevention are often insufficient (30). Schools without policies and programs to address childhood obesity may be fostering an environment in which unhealthy habits are acceptable among youngsters. Bastida et al. (31) assert that comprehensive school health programs are essential for modifying children’s behaviors and enhancing their overall health outcomes. The availability of physical activity programs, even if not universally implemented, suggests that there is potential for schools to incorporate more structured and comprehensive obesity prevention measures into their daily routines.

The current study also identified major obstacles encountered by teachers in the prevention and management of childhood obesity including the lack of resources, unavailability of training, and insufficient time. These constraints correspond with prior research in other contexts that emphasize structural challenges, such as insufficient resources and training, as significant impediments to teachers’ successful participation in health promotion initiatives (30, 32). This is unsurprising, considering that school-based policies and programs have not been widely implemented in low-and middle-income countries and therefore the required resources and training that would be part of such programs are not adequately provided. The data clearly indicates the necessity for professional development and organized programs for teachers to improve their understanding and ability to address childhood obesity. Providing adequate time for health education and physical activity, together with professional training, were considered essential facilitators for teachers’ involvement in obesity prevention initiatives. This indicates that, given appropriate assistance and resources, teachers are prepared and capable of supporting the prevention and treatment of childhood obesity within educational institutions. However, parents’ involvement cannot be underestimated, as dietary and lifestyle modifications have been shown to reduce childhood obesity. Teachers’ knowledge and parents’ involvement are therefore key facilitators to the prevention and management of childhood obesity.

Teachers’ awareness status, along with various factors such as social economic influence, types of challenges faced by teachers, physical education practices, health screening practices, healthy eating education practices, and counseling practices, demonstrated statistically significant relationships with the prevention and management of childhood obesity. These associations suggest that both knowledge and practical implementation of health-promoting strategies are key components influencing teachers’ engagement in obesity-related initiatives. Conversely, age, gender, and educational level were found to be statistically non-significant, indicating that demographic characteristics may play a limited role in influencing teachers’ participation in such programs. As such, all teachers have the potential to lead such prevention and management measures. Subsequently, a lack of statistically significant predictors of teachers’ participation in childhood obesity prevention programs highlights the complexity of engaging teachers in these initiatives, as numerous factors may influence participation without achieving the desired threshold for statistical significance (33). Teachers who regularly participated in health education or physical activity programs demonstrated a higher likelihood of involvement, suggesting the influence of continuous engagement with health-related curricula and physical education initiatives.

### Strengths, limitations and future directions

This study provides key findings into the role of teachers in preventing and managing childhood obesity, offering insights that are valuable for both individual and institutional stakeholders. However, these insights are tempered by a number of limitations. The study is limited to a single municipality and public schools in an urban area, which constrains the applicability of its findings to other districts, rural areas, or private schools, limiting the study’s generalizability and reducing the relevance for broader policy or program design. The convenience sampling technique employed may have introduced a risk of selection bias, which may also limit the generalizability of the findings to the broader population. Overlapping predictors, such as physical activity and healthy eating education, may obscure independent effects due to their interrelated nature, resulting in non-significant results in the adjusted logistic regression model. Additionally, measurement errors from self-reported data may have introduced bias, weakening associations. Moreover, contextual barriers, including socioeconomic challenges, lack of training, and resource limitations, may have overshadowed individual predictors, highlighting the need for larger, more robust studies with objective measures and context-specific analyses to further strengthen these findings.

Future research should include multiple districts encompassing both urban and rural settings across the country, as well as private and public schools to enhance the generalizability of findings and provide more comprehensive evidence to guide national policy and program development. Additionally, longitudinal and intervention-based research could evaluate the effectiveness of teacher-led obesity prevention programs, while examining the impact of comprehensive training, parental engagement, and policy implementation on children’s long-term health and behavioral outcomes.

## Conclusion

Teachers showed general awareness of childhood obesity but limited understanding of its public health impact. Gender disparities, inadequate institutional support, and resource constraints hinder effective engagement. Findings also revealed deficiencies in school policies, low access to preventive programs, and inadequate training, stressing the necessity to strengthen both education and healthcare systems in Ghana. Targeted teacher training, adequate institutional support, and stronger school-community collaborations should be prioritized to enhance teachers’ effectiveness in the prevention and management of childhood obesity in Ghana. The study offers actionable recommendations to policymakers, educators, and school administrators, highlighting the critical role of incorporating structured health curricula, increasing opportunities for physical exercise, and partnering with healthcare professionals to sustain childhood obesity prevention efforts.

## Data Availability

The original contributions presented in the study are included in the article/Supplementary material, further inquiries can be directed to the corresponding author.
