# Supplementary material for: The role of teachers in the prevention and management of childhood obesity among school-aged children in Ghana: a cross-sectional study
Source: Front Public Health. 2025 Dec 3;13:1724617. doi: 10.3389/fpubh.2025.1724617 (PMC12708575; doi:10.3389/fpubh.2025.1724617)
Supplement: Supplementary file 1 [file Table_1.DOCX]

**DATA COLLECTION TOOL/ QUESTIONNAIRE**

KWAME NKRUMAH UNIVERSITY OF SCIENCE AND TECHNOLOGY

SCHOOL OF PUBLIC HEALTH

DEPARTMENT OF GLOBAL HEALTH & NON-COMMUNICABLE DISEASES


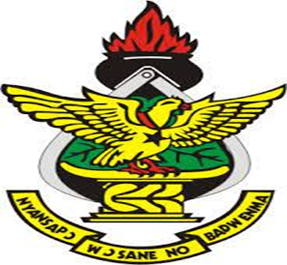


TOPIC: **Assessment of the role of teachers in prevention and management of childhood obesity among school aged children in Oforikrom** **Municipality.**

DATE NAME OF SCHOOL

........................................ ................................................ QUESTIONNAIRE NO GRADE LEVEL

………………………….. …………………………..

Dear participant,

This research is being conducted by a student from Kwame Nkrumah University of Science and Technology, School of Public Health. The purpose of this study is to assess the role of teachers in prevention and management of childhood obesity among school aged children in Oforikrom municipality. I therefore encourage each participant to kindly respond to the questions with appropriate and valid answers to ensure accuracy and correct representation. No one will ask you to write your name on any part of the questionnaire. Data analysis will group all participants to protect anonymity, and the findings will be used to improve childhood obesity in Oforikrom municipality. Your confidentiality and anonymity are assured in this study. If you agree to participate, kindly answer the questions on this survey. Thank you.

**SECTION A: DETAILS ON DEMOGRAPHIC CHARACTERISTICS OF PARTICIPANTS**

1. Age, tick in the space provided that is corresponding your age range:
2. 20-30 []
3. 31-40 []
4. 41-50 []
5. 51 and above []
6. What is your gender? please tick where applies:
7. Male []
8. Female []
9. Which level of education do you have?
10. Diploma []
11. Bachelor’s degree []
12. Master’s degree []
13. Others (specify):

................................................................................................................................

1. How many Years of Teaching experience do you have?
2. 0-5 years []
3. 6-10 years []
4. 11-15 years []
5. 16 years and above []
6. What grade levels are you currently teaching?
7. Lower primary (grades 1-3) []
8. Upper primary (grades 4-6) []
9. Junior high school (grades 7-9) []

**SECTION B: AWARENESS AND PERSPECTIVES OF PARTICIPANT ABOUT CHILDHOOD OBESITY**

6. How familiar are you with the issue of overweight and obesity in the general population?

- 1. extremely familiar []
  2. familiar []
  3. familiar to some extent []
  4. Not familiar []

7. How familiar are you with the issue of childhood obesity?

- 1. Extremely familiar
  2. familiar []
  3. familiar to some extent []
  4. Not familiar []

8. How familiar are you with the issue childhood obesity, it’s causes, health implications and prevention measures?

- 1. Extremely familiar []
  2. familiar []
  3. familiar to some extent []
  4. Not familiar []

9. If you are familiar with childhood obesity, its causes, health implications and prevention measures answer the following questions (tick where applies).

A) Causes of childhood obesity

i. Childhood obesity is caused by unhealthy eating habit.

- 1. Yes []
  2. No []

ii. Childhood obesity is due to lack of physical exercise.

- 1. Yes []
  2. No []

iii. Childhood obesity is caused by genetic predisposition.

- 1. Yes []
  2. No []

iv. Childhood obesity is influenced by socio-economic status.

B) Health implications

v. Childhood obesity increases the risk of developing type 2 diabetes.

- 1. Yes []
  2. No []

vi. Childhood obesity is associated with blood pressure and heart disease.

- 1. Yes []
  2. No []

vii. Childhood obesity is associated with psychological difficulties and low self esteem.

- 1. Yes []
  2. No []

C) Prevention of childhood obesity

viii. Childhood obesity can be prevented by regular physical activity.

- 1. Yes []
  2. No []

x. Childhood obesity can be prevented by promoting healthy eating habit.

- 1. Yes []
  2. No []

10. How did you learn about childhood obesity?

- 1. School []
  2. Church []
  3. Hospital []
  4. Durbar []
  5. Newspaper []
  6. Social media []
  7. Others (specify):

...............................................................................................................................

11. Do you think childhood obesity is a major problem in Kumasi?

- 1. Yes []
  2. No []
  3. Not sure []

12. How would you rate the level of the problem in Kumasi?

- 1. severe []
  2. Moderate []
  3. Low []

13. Who should play a leading role in addressing childhood obesity select up to three?

- 1. Parents []
  2. Doctor []
  3. Nurses []
  4. Teachers []
  5. Public health officers []
  6. Community leaders []
  7. Policy makers []
  8. Others (specify):

.........................................................................................................................

14. What are the measures for addressing childhood obesity are you aware of?

- 1. Physical activity []
  2. Healthy eating habit []
  3. Health education []

1. Supportive environment for tackling childhood obesity []
2. Others (specify)

.........................................................................................................................

15. Should teachers be involved in preventing and treating childhood obesity?

1. Yes []
2. No []
3. Not sure []

16. What should they be involved in?

1. Health education []
2. Physical activity []
3. Others (specify):

...........................................................................................................................

17. How important is the role of teachers in addressing childhood obesity?

i. Extremely important []

j. Important []

k. Important to some extent []

l. Not important []

**SECTION C: INSTITUTIONAL PRACTICES**

1. Do you know of any school-wide programs/initiatives to prevent childhood obesity?
2. Yes []
3. No []
4. Not sure []
5. Are there programmes in your school/ curriculum regarding physical activity?
6. Yes []
7. No []
8. Not sure []
9. Are there programmes in your school/ curriculum regarding healthy diets?
10. Yes []
11. No []
12. Not sure []
13. Do you agree that school meals follow nutritional guidelines to help reduce childhood obesity?
14. Agree []
15. Strongly agree []
16. Disagree []
17. Strongly disagree []
18. Not applicable []
19. Does your school have any initiatives/programs to promote healthy snacks and beverages?
20. Yes []
21. No []
22. Not sure []
23. Do educational and awareness programs about diabetes exist in your school?
24. Yes []
25. No []
26. Not sure []
27. How frequently are the following measures for addressing childhood obesity practiced in your school?

|  | Always | Frequently | Sometimes | Rarely | Never |
| --- | --- | --- | --- | --- | --- |
| Physical education sessions |  |  |  |  |  |
| Outdoor play |  |  |  |  |  |
| Education on healthy eating |  |  |  |  |  |
| Health screening of students |  |  |  |  |  |
| Counselling of students |  |  |  |  |  |
| Sharing of health education materials |  |  |  |  |  |
| Discussing student’s health with parents |  |  |  |  |  |
| Health education sessions for parents |  |  |  |  |  |
| Education for teachers and students by health professionals |  |  |  |  |  |

**SECTION D: EXISITING PRACTICES OF TEACHERS**

1. How frequently do you have physical education classes with your students?
   - - 1. Daily []
       2. Several times a week []
       3. Once a week []
       4. Once a month []
       5. Rarely []
       6. Never []

26. How frequently do you educate your students on healthy eating?

1. Daily []
2. Several times a week []
3. Once a week []
4. Once a month []
5. Rarely []
6. Never []
7. Which of these do you promote the most?

m. Healthy eating []

n. Regular exercise []

o. Others (specify):

....................................................................................................................................

1. Do you engage with parents and guardians to encourage regular exercise?
2. Yes []
3. No []

28. If yes, how If yes, how frequently do you do the above?

1. Daily []
2. Several times a week []
3. Once a week []
4. Once a month []
5. Rarely []
6. Never []
7. Do you do you engage with parents and guardians in promoting healthy eating at home?
8. Yes []
9. No []
10. Sometimes []

30. If yes, how frequently do you do the above?

- - 1. Daily []
    2. Several times a week []
    3. Once a week []
    4. Once a month []
    5. Rarely []
    6. Never []

1. Have you taken part in any parent meetings where the topics of child’s health and obesity prevention have been discussed?

- - 1. Yes []
    2. No []

1. Do you agree that the school could involve parents in encouraging healthy lifestyle at home in any other ways?

i. Agree []

j. Strongly agree []

k. Disagree []

l. Strongly disagree []

33. Do you have the training necessary to assist you in promoting the prevention of childhood obesity in your classroom?

1. Yes []
2. No []

34. Do you have the material or tools necessary to assist you in promoting the prevention of childhood obesity?

1. Yes []
2. No []

35. What tools are needed to assist you in promoting childhood obesity in your classroom?

1. Nutrition education programmes []
2. Physical activity programmes []
3. Access to healthy food options []
4. Others (specify):

...............................................................................................................................................

36. How certain are you that you can contribute to changing the eating habits of your students?

1. Very confident []
2. Confident []
3. Somehow confident []
4. Not confident []

37. How certain are you that you can contribute to changing the exercise habits of your students?

- 1. Very confident []
  2. Confident []
  3. Somehow confident []
  4. Not confident []

38. What will you recommend that teachers do to help in preventing and controlling childhood obesity?

- 1. Increase health education in their teaching []
  2. promote physical activity programmes []
  3. Engage and counsel students []
  4. Engage parents []
  5. Advocate for policies and curricula improvements with school administration []
  6. Others (specify):

..............................................................................................................................

**SECTION E: CHALLENGES IN PREVENTION AND MANAGEMENT OF CHILDHOOD OBESITY**

39. Do you face any challenges regarding playing a role in the prevention of childhood obesity in your students?

1. Yes []
2. No []

40. What challenges do you face in your school regarding prevention of childhood obesity?

1. inadequate resources []
2. No training []
3. Not enough time []
4. There is no support from parents []
5. No school administration support []
6. Others (specify)

........................................................................................................................................

41. How significantly do the challenges hinder you from playing a role in the prevention of childhood obesity?

1. Extremely significant []
2. Significant []
3. Significant to some extent []
4. Not significant []

42. Do you face any challenges regarding the management of childhood obesity among your students?

1. Yes []
2. No []

43. What challenges do you face in your school regarding the management of overweight in your class? (tick all that applies)

1. Inadequate resources []
2. No training []
3. Not enough time []
4. There is no support from parents or guardians []
5. No school administration support []
6. No guidance []
7. No permission to discuss the topic []
8. Others (specify)

...........................................................................................................................

44. How significant do the challenges hinder you from playing a role in the management of childhood obesity in your class?

1. Extremely significant []
2. Significant []
3. Significant to some extent []
4. Not significant []

45. What are the roles of facilitators in prevention and management of childhood obesity?

1. High []
2. Moderate []
3. Low []

**SECTION F:** **POLICIES AND INSTITUTIONAL FRAMEWORKS SUPPORTING THE ROLE OF TEACHERS**

46. Are there any policies, plans or guidelines in your school for preventing and managing childhood obesity?

- - 1. Yes []
    2. No []
    3. Not sure []

47. Are there any policies or guidelines in your school supporting the role of teachers in preventing and managing childhood obesity?

- - 1. Yes []
    2. No []
    3. Not sure []

48. Are there any policies or guidelines outside your school (such as education or municipal offices) supporting the role of teachers in preventing and managing childhood obesity?

- - 1. Yes []
    2. No []
    3. Not sure []

49. Is there any institutional arrangement in your school that supports the role of teachers in preventing and managing childhood obesity?

- - 1. Yes []
    2. No []
    3. Not sure []

50. Are there any resources at your school that you can make use of to support your role in preventing and managing childhood obesity?

- - 1. Yes []
    2. No []
    3. Not sure []

51. If yes, what kind of resources are available?

- - 1. Training provision []
    2. Access to teaching curricular []
    3. Access to teaching materials []
    4. Working with health care providers []
    5. Engagement with parents or guardians []
    6. Others (specify):

..................................................................................................................................................

52. Are there any resources from Ministry of Education that you can make use of to support your role in preventing and managing childhood obesity?

- - 1. Yes []
    2. No []
    3. Not sure []

53. What type of resources are available from the Ministry of Education?

.......................................................................................................................................

54. What kind of policies do you think Ministry of Health and Education should put in place to reduce childhood obesity? (tick all that applies)

- - 1. Healthy school meal programmes []
    2. Physical activity programmes []
    3. Health education []
    4. Health screening []
    5. Others (specify):

......................................................................................................................................................

.............................................................................................................................

55. What kind of assistance or materials would you need to better combat childhood obesity in your school? (tick all that apply)

- - 1. Training provision []
    2. Access to teaching curricular []
    3. Enough time for physical activity []
    4. Working with health care providers []
    5. Participation of parents or guardians []
    6. Others (specify):

...........................................................................................................................

56. what do you think should the administration of the school can do to help manage and prevent childhood obesity at your school?

- - 1. Incorporate more health education content into the curriculum []
    2. Provide an environment that is conducive to physical activity []
    3. Increase time for physical activity sessions []
    4. Access to healthy food options []
    5. Give teachers resources and training []
    6. Implement health policies for the entire school []
    7. Provide counselling []
    8. Involve parents and guardians []
    9. Involve the community []

vi. Others (specify): ....................................................................................
